# Erythromélalgie: complication exceptionnelle de la polyarthrite rhumatoïde

**DOI:** 10.11604/pamj.2016.25.226.9612

**Published:** 2016-12-07

**Authors:** Zeineb Alaya, Walid Osman

**Affiliations:** 1 Service de Rhumatologie, Hôpital Farhat Hached, Sousse, Tunisie; 2 Service d’Orthopédie, Hôpital Sahloul, Sousse, Tunisie

**Keywords:** Erythromélalgie, polyarthrite rhumatoïde, Tunisie

## Image en médecine

Il s’agit d’une patiente âgée de 53 ans, suivie pour une polyarthrite rhumatoïde (PR) érosive très sévère avec luxation atloido-axoidienne sous anti TNFα. Elle a consulté pour des crises douloureuses à type de brûlure au niveau des mains, déclenchées par la chaleur et soulagées par le froid, évoluant depuis 2 semaines, concomitantes à une poussée de sa PR. L’examen dermatologique durant la crise, a révélé un érythème douloureux palmaire bilatéral, intéressant surtout les pulpes digitales et les éminences thénar et hypothénar avec rougeur et chaleur locale. Le diagnostic d’érythromélalgie (EM) a été retenu. Dans le cadre de l'enquête étiologique, nous avons éliminé les principales causes d'EM secondaire: prise médicamenteuse, un lupus, un syndrome myéloprolifératif, une néoplasie, un diabète, une prise de toxique, une virose ou une hyperthyroïdie à l'origine de cet acrosyndrome. La PR était à l'origine de l'EM secondaire dans notre cas, ce qui est exceptionnel au cours ce rhumatisme. Le pronostic dépend de la sévérité de la PR et de sa réponse au traitement. La patiente a eu un switch vers le tocilizumab.

**Figure 1:**
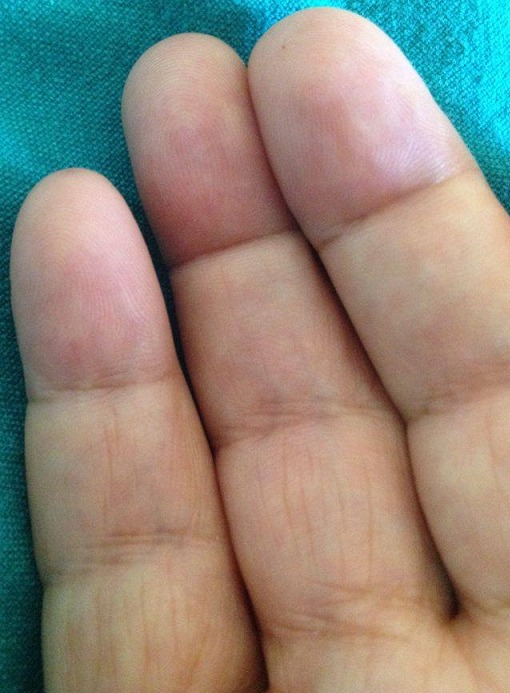
Érythème palmaire bilatéral avec rougeur secondaire à une érythromélalgie chez une patiente suivie pour une polyarthrite rhumatoïde

